# A Happy Home

**Published:** 1879-01

**Authors:** 


### A HAPPY HOME.

There is no better way, no safer way, no easier way, no more certain way to save children from the debasing influences of the street, of doubtful associations and dangerous resorts, than to make home attractive. This should be made the intelligent and conscientious study of every parent who has a due regard for the future welfare and usefulness of sons and daughters.

From eight to sixteen, said an eminent English Judge, is the time during which nine-tenths of the criminals who came before me have cast their destinies for life. It is from

"EIGHT TO SIXTEEN"

That parents should give special attention to weave around their children the web which shall keep them at home, in their presence and near their persons, by affectionate ways, by kindly treatment, by loving words, by warning sympathies, by intelligent forbearances, by generous allowances. In addition to this, safe, profitable, instructive and interesting reading should be provided for them; than which nothing is more appropriate than *Hall's Journal of Health.*

Delicious Sleep comes only to the young and the day-laborer. Few who live indoors, or who make their living by brain-work, sleep well. One reason is, they do not ascertain how much sleep they require. We all get up in the morning with a certain and varying amount of energy, strength or vital power; and the sleep we require during the night is exactly proportioned to the energy expended during the day, in thought, or work, or mere exercise, and nature will give no more sleep than will make up that supply. And yet, there are intelligent men who will sit around the house all day, as on a rainy Sunday; will go to bed an hour or two earlier, and because they do not sleep the night through, complain of not sleeping well, and begin to think of taking opiates, chloral, or other form of anodyne, which never give natural, restful sleep, and always leave the system in a deranged condition.

Another case: A man goes to bed regularly at ten o'clock all the year round. In Summer time he wakes at daylight—about five in the morning—and all is well. But when Winter comes, and he wakes two or three hours before day, and can't go to sleep again, he begins to grow nervous, to think something is going wrong, and talks about taking medicine, forgetting that in Winter daylight comes several hours later and that he really sleeps as long as he did in Summer.

There is an instinct in nature which prompts an awaking in the morning when sleep enough has been had to supply the energy expended during the day—a self-acting mechanism—one of the most wonderful in the human system, and yet we think so little of it. If a man wants to sleep the whole night through, wants to sleep an hour or two or three longer, he has only to work harder and a few hours longer in the open air; that is nature's anodyne.

---

Music "hath charms to sooth the savage breast" as well to-day as in David's time, who handled the harp so skilfully that he drove the demons of sin from the heart and head of King Saul. It is safe to say, that every one who has an ear for music, vocal or instrumental, should cultivate it to the fullest extent possible, for its practice develops the lungs, improves the health, elevates the tastes, purifies the affections

